# Estimating the excess burden of pertussis disease in Australia within the first year of life, that might have been prevented through timely vaccination

**DOI:** 10.1093/ije/dyac175

**Published:** 2022-09-13

**Authors:** Duleepa Jayasundara, Deborah Randall, Sarah Sheridan, Vicky Sheppeard, Bette Liu, Peter C Richmond, Christopher C Blyth, James G Wood, Hannah C Moore, Peter B McIntyre, Heather F Gidding

**Affiliations:** NSW Biostatistics Training Program, NSW Ministry of Health, St Leonards, NSW, Australia; Women and Babies Research, Kolling Institute, Northern Sydney Local Health District, St Leonards, NSW, Australia; University of Sydney, Northern Clinical School, St Leonards, NSW, Australia; Women and Babies Research, Kolling Institute, Northern Sydney Local Health District, St Leonards, NSW, Australia; University of Sydney, Northern Clinical School, St Leonards, NSW, Australia; Women and Babies Research, Kolling Institute, Northern Sydney Local Health District, St Leonards, NSW, Australia; University of Sydney, Northern Clinical School, St Leonards, NSW, Australia; National Centre for Immunisation Research and Surveillance of Vaccine Preventable Diseases, Sydney, NSW, Australia; Public Health Unit, South Eastern Sydney Local Health District, Sydney, NSW, Australia; School of Public Health, University of Sydney, Sydney, NSW, Australia; National Centre for Immunisation Research and Surveillance of Vaccine Preventable Diseases, Sydney, NSW, Australia; School of Population Health, UNSW Medicine, UNSW, Sydney, NSW, Australia; Wesfarmers Centre of Vaccines and Infectious Diseases, Telethon Kids Institute, University of Western Australia, Perth, WA, Australia; Department of General Paediatrics, Perth Children's Hospital, Perth, WA, Australia; School of Medicine, University of Western Australia, Perth, WA, Australia; Wesfarmers Centre of Vaccines and Infectious Diseases, Telethon Kids Institute, University of Western Australia, Perth, WA, Australia; School of Medicine, University of Western Australia, Perth, WA, Australia; Department of Infectious Diseases, Perth Children's Hospital, Perth, WA, Australia; Department of Microbiology, PathWest Laboratory Medicine WA, Perth Children's Hospital, Perth, WA, Australia; School of Population Health, UNSW Medicine, UNSW, Sydney, NSW, Australia; Wesfarmers Centre of Vaccines and Infectious Diseases, Telethon Kids Institute, University of Western Australia, Perth, WA, Australia; National Centre for Immunisation Research and Surveillance of Vaccine Preventable Diseases, Sydney, NSW, Australia; Department of Women’s and Children’s Health, Dunedin School of Medicine, University of Otago, Dunedin, New Zealand; Women and Babies Research, Kolling Institute, Northern Sydney Local Health District, St Leonards, NSW, Australia; University of Sydney, Northern Clinical School, St Leonards, NSW, Australia; National Centre for Immunisation Research and Surveillance of Vaccine Preventable Diseases, Sydney, NSW, Australia; School of Population Health, UNSW Medicine, UNSW, Sydney, NSW, Australia

**Keywords:** Pertussis, propensity score weighting, vaccination timeliness

## Abstract

**Background:**

Previous Australian studies have shown that delayed vaccination with each of the three primary doses of diphtheria-tetanus-pertussis-containing vaccines (DTP) is up to 50 % in certain subpopulations. We estimated the excess burden of pertussis that might have been prevented if (i) all primary doses and (ii) each dose was given on time.

**Methods:**

Perinatal, immunization, pertussis notification and death data were probabilistically linked for 1 412 984 infants born in two Australian states in 2000–12. A DTP dose administered >15 days after the recommended age was considered delayed. We used Poisson regression models to compare pertussis notification rates to 1-year of age in infants with ≥1 dose delayed (Aim 1) or any individual dose delayed (Aim 2) versus a propensity weighted counterfactual on-time cohort.

**Results:**

Of all infants, 42% had ≥1 delayed DTP dose. We estimated that between 39 to 365 days of age, 85 (95% CI: 61–109) cases per 100 000 infants, could have been prevented if all infants with ≥1 delayed dose had received their three doses within the on-time window. Risk of pertussis was higher in the delayed versus the on-time cohort, so crude rates overestimated the excess burden (110 cases per 100 000 infants (95% CI: 95–125)). The estimated dose-specific excess burden per 100 000 infants was 132 for DTP1, 50 for DTP2 and 19 for DTP3.

**Conclusions:**

We provide robust evidence that improved DTP vaccine timeliness, especially for the first dose, substantially reduces the burden of infant pertussis. Our methodology, using a potential outcomes framework, is applicable to other settings.

Key Messages

- Receipt of vaccines at the recommended age (on-time) is suboptimal in many countries, but whether this contributes to the burden of pertussis in infants is not well studied.
- Using individual-level linked population data and applying propensity weighting to assemble counterfactual on-time cohorts, we estimated that on-time vaccination of 1175 infants with at least one delayed dose prevents one case of pertussis by 1 year of age.
- The greatest reductions were seen for receipt of the first dose (due at 2 months), and in sub-populations with higher baseline rates of pertussis and with longer delays.
- The study provides robust methods to estimate the contribution of delayed infant pertussis vaccinations to pertussis incidence, which can be applied in other settings.

## Introduction

Pertussis (whooping cough) continues to cause significant morbidity and mortality, especially in young children. Recent modelling estimated that, globally, 24 million cases and 161 000 associated deaths occurred in children <5 years of age in 2014, with the greatest burden of severe disease in infants <1 year of age.^1^ The highest incidence is in low- and middle-income countries, but many high-income countries, including Australia, have recently experienced a resurgence despite long-standing vaccination programmes achieving coverage of over 90% by 1 year of age.^2–4^ However, coverage at this traditional milestone can mask substantial disparities in on-time vaccine uptake. We have previously reported on-time coverage (within 30 days of the recommended due date) with each infant dose of diphtheria-tetanus-pertussis (DTP) vaccine in Australia (due at 6–8 weeks and 4 and 6 months of age) as low as 50–60% in some population groups.^5^ Infants with multiple older siblings, infants born preterm and Aboriginal and/or Torres Strait Islander (herein respectfully referred to as Aboriginal) infants, and infants born to young mothers, mothers who smoked during pregnancy, or mothers from the Oceania region (excluding Australia), were most at risk of delayed vaccination.^6^ Similarly, low on-time coverage rates have also been reported in other countries such as the USA and in Europe.^7–9^

Delays in vaccination result in delayed protection against pertussis at both individual and community levels. However, few studies have quantified how much the residual burden of pertussis could be reduced by improving vaccination timeliness.^10–13^ Furthermore, no studies to date have used individual-level linked vaccination and pertussis case notifications to enable accurate assessment of population impact. We aimed to estimate the excess pertussis burden in Australian children within the first year of life, using a novel population-based cohort approach. Aim 1 estimated the excess burden of pertussis that may have been prevented had all three primary doses of DTP been given on time among a cohort with at least one delayed dose, and Aim 2 estimated the dose-specific excess burden that may have been prevented if each dose and all subsequent doses were received on time.

## Methods

### Data sources

Perinatal and birth records for infants born in New South Wales (NSW) and Western Australia (WA) from 2000 to 2012 were probabilistically linked, using personal identifiers, to immunization, death registrations and pertussis notification records available up to 31 December 2013. The birth cohort accounted for approximately 40% of all Australian births. The perinatal data included maternal and child demographic characteristics, maternal medical and obstetric history and information on the labour and delivery of all births. Birth registration records included demographic details on both parents and the infant. The Aboriginal status of each infant was derived by applying an algorithm using all linked datasets except deaths.^14^ Vaccination records were obtained from the Australian Childhood Immunization Register (ACIR, now known as the Australian Immunization Register), which included details (type, date of administration) of all vaccinations given to children <7 years of age. Pertussis notification records were obtained from the NSW and WA disease notification registers, which require mandatory reporting of laboratory-confirmed cases under state-based legislation. Full details on the data linkage process and assembly of the cohort are provided elsewhere.^15^^,^^16^

### Study outcome

The outcome of interest for each infant was the first episode of notified pertussis^17^ occurring during follow-up to 1 year of age.

### Definitions for dose-specific vaccination status

Age at immunization was calculated using the date of immunization recorded in ACIR and the date of birth recorded in perinatal data. The Australian vaccination schedule considers children due for their first three doses of DTP at 2 months (61 days; DTP1), 4 months (122 days; DTP2), and 6 months of age (183 days; DTP3).^18^ Definitions of dose-specific on-time and delayed windows, consistent with Gidding *et al.* (2020),^6^ are shown in Table 1.

**Table 1:** Dose-specific vaccination status based on age at immunization

Vaccination status	DTP1 Recommended at 2 months^a^ (61 days)	DTP2 Recommended at 4 months (122 days)	DTP3 Recommended at 6 months (183 days)
Invalid	<39 days	<108 days	<169 days
On-time	39 days—76 days	108 days—137 days	169 days—198 days
Delayed	>76 days or not received	>137 days or not received	>198 days or not received

DTP, diphtheria-tetanus-pertussis vaccine.

a DTP1 is recommended as early as 6 weeks (42 days) of age, and New South Wales actively promoted this recommendation from 2009.^22^

### Inclusion and exclusion criteria

For Aims 1 and 2, only infants with both a perinatal and a birth registration record (97.5% of live births in the perinatal dataset), who were the firstborn child of each pregnancy, were alive at 39 days of age and had not received DTP1 or been notified with pertussis before then, were included. Infants with suspected errors in their vaccination records or who had no DTP1, 2 or 3 recorded but had a subsequent dose recorded (suggesting incomplete recording) were excluded. For Aim 2, only the subset of infants who were eligible to receive the dose under consideration ‘on-time’ and who had not been notified with pertussis before then were included (see Supplementary Methods, available as Supplementary data at *IJE* online for details).

### Analysis

#### Propensity weighting of on-time cohorts

The on-time and delayed cohorts, assembled for both Aims 1 and 2, showed significant differences in the distribution of potential confounders that have previously been identified as strong predictors of delayed vaccination^6^ and in demographic characteristics (Table 2; Supplementary Table S1, available as Supplementary data at *IJE* online). To control for these potential confounders, we created a counterfactual on-time cohort by applying stabilized weights based on inverse probability of treatment (IPT) methodology (see Supplementary Methods).^19^ This process enabled application of a potential outcomes framework to assess the average treatment effect in the untreated (ATU), i.e. how the rate of pertussis, on average, would differ in the delayed cohort if, counter to the fact, they had been vaccinated on time.^20^

**Table 2:** Characteristics of the on-time and delayed study cohorts (Aim 1)

Characteristic	On-time (*n* = 820 001)	Delayed (*n* = 592 983)
Gestational age		
<=28 weeks	0.16%	0.37%
29–32 weeks	0.60%	0.89%
33–36 weeks	4.96%	5.51%
37–40 weeks	78.25%	77.94%
>40 weeks	16.02%	15.27%
Missing	0.01%	0.02%
Parity		
0	48.03%	27.98%
1–2	49.04%	63.87%
3+	2.84%	8.04%
Missing	0.09%	0.11%
Aboriginal status		
Non-Aboriginal	96.76%	92.90%
Aboriginal	3.24%	7.10%
Missing	0.00%	0.00%
Year of birth		
2000	7.00%	7.71%
2001	7.06%	7.41%
2002	7.04%	7.41%
2003	6.85%	7.76%
2004	6.85%	7.68%
2005	7.29%	7.89%
2006	7.81%	7.79%
2007	8.10%	8.12%
2008	8.34%	7.85%
2009	8.43%	7.62%
2010	8.38%	7.72%
2011	8.54%	7.61%
2012	8.30%	7.43%
Season of birth		
Spring	25.75%	24.53%
Summer	24.89%	25.80%
Autumn	25.16%	26.00%
Winter	24.21%	23.67%
SEIFA quintile^a^		
91–100%	9.23%	8.72%
76–90%	15.78%	14.06%
26–75%	48.43%	46.18%
11–25%	14.74%	15.86%
0–10%	9.29%	11.89%
Missing	2.53%	3.27%
Mother's age		
>=35 years	20.64%	21.85%
30–34 years	33.81%	31.36%
25–29 years	28.95%	26.61%
20–24 years	13.28%	15.42%
<20 years	3.32%	4.76%
Smoking status during pregnancy		
No	89.31%	82.00%
Yes	10.47%	17.82%
Missing	0.21%	0.19%
Remoteness category^b^		
Major cities	75.67%	71.90%
Inner regional	14.80%	16.04%
Outer regional	5.63%	6.78%
Remote	1.56%	2.23%
Missing	2.34%	3.05%
Mother's region of birth		
Australia	67.52%	68.86%
Oceania	3.02%	5.04%
Europe	6.05%	6.34%
Middle East and Africa	5.37%	5.14%
Asia	13.69%	9.62%
America	1.36%	1.62%
Missing	2.99%	3.39%
State of birth registration		
Western Australia	22.24%	26.75%
New South Wales	77.76%	73.25%

a SEIFA, socioeconomic indexes for areas. Measured using the Index of Relative Socio-economic Disadvantage (IRSD).^23^

b Measured using the Accessibility/Remoteness Index of Australia (ARIA).^24^

## Aim 1-specific analysis methods

### Main analysis

In the Aim 1 analysis, infants’ vaccination, first pertussis notification and death events were observed from 39 days of age to the earliest of the following: first invalid DTP dose, death, fourth DTP dose or 1 year of age (i.e. the follow-up period). The exposure cohorts were then defined as:

- the ‘on-time’ cohort—infants who received all DTP doses within the follow-up period on time;
- the delayed cohort—infants who did not receive all three DTP doses on time (at least one DTP dose delayed or not received within the follow-up period).

If the date of death was within the on-time window of a dose, the exposure status was based only on prior doses.

Poisson regression models with robust error variances were fitted without and with stabilized IPT weights to obtain crude and weighted incidence rates of pertussis notification (see Supplementary Methods for details).

### Sub-analyses

We conducted three sub-analyses. First, we evaluated the same measures of effect in subgroups that are known to have high rates of delayed vaccination compared with the general population (Table 3).^6^ This was to assess the specific excess burden and number needed to vaccinate on time in each high-risk group. Second, we restricted the delayed cohort to infants who received at least one dose prior to turning 1 year of age for comparison with the on-time cohort. This was to assess the excess burden in those who may be more likely to engage with health services and thus respond to interventions to improve timeliness. Third, the measures of effect were stratified by calendar year of birth to assess the difference in excess burden during years of high versus low pertussis incidence.

**Table 3:** Unweighted and weighted rates of pertussis and epidemiological measures of the excess burden: Aim 1 analysis

Population subgroup	Rate of pertussis in delayed cohort^a^ ($I_{D}$)	Unweightedrate of pertussis in on-time cohort^a^	Weighted rate of pertussis in on-time cohort^a^^,^^b^ ($I_{o}$)	Relative rate^c^$\left(\frac{I_{D}}{I_{O}}\right)$	Excess cases^a^^,^^c^$\left(I_{D}-I_{O}\right)$	Number needed to vaccinate on time to prevent one case^c^$\left(\frac{100,000}{I_{D}-I_{O}}\right)$	Attributable fraction (%)^c^$\left[\left(1-\frac{I_{o}}{I_{D}}\right)\times 100\%\right]$
All groups	247 (234, 259)	137 (129, 145)	162 (150, 173)	1.53 (1.40, 1.66)	85 (68, 102)	1175 (942, 1408)	34 (29, 40)
Birth registered in NSW	262 (247, 277)	150 (140, 160)	176 (163, 190)	1.49 (1.35, 1.63)	86 (66, 106)	1168 (894, 1442)	33 (26, 39)
Birth registered in WA	205 (182, 227)	92 (78, 106)	116 (95, 137)	1.76 (1.39, 2.13)	88 (58, 119)	1131 (739, 1523)	43 (31, 55)
Non-Aboriginal	229 (216, 242)	131 (123, 139)	150 (140, 161)	1.52 (1.39, 1.66)	79 (62, 95)	1271 (1006, 1536)	34 (29, 40)
Aboriginal	479 (413, 545)	321 (251, 391)	315 (229, 401)	1.52 (1.05, 1.98)	163 (55, 272)	612 (206, 1019)	34 (14, 54)
Gestational age <=36 weeks	335 (279, 392)	193 (152, 233)	265 (193, 337)	1.26 (0.86, 1.67)	70 (-22, 162)	1426 (-442, 3294)	21 (-04, 46)
Parity >=3	486 (423, 548)	327 (252, 402)	322 (242, 403)	1.51 (1.08, 1.93)	163 (61, 265)	613 (229, 996)	34 (15, 52)
Mother born in Oceania excluding Australia	285 (225, 346)	155 (105, 205)	166 (106, 225)	1.72 (1.00, 2.44)	120 (35, 204)	836 (243, 1430)	42 (18, 66)
Mother aged <20 years	348 (279, 417)	231 (173, 290)	248 (178, 317)	1.41 (0.92, 1.89)	100 (3, 198)	995 (28, 1963)	29 (04, 53)
Mother smoked during pregnancy	315 (281, 349)	188 (158, 217)	199 (162, 237)	1.58 (1.24, 1.92)	116 (65, 166)	865 (488, 1243)	37 (23, 50)
At least one dose within the first year of life	228 (215, 241)	137 (129, 145)	163 (152, 174)	1.40 (1.28, 1.53)	65 (48, 82)	1533 (1131, 1935)	29 (22, 35)

NSW, New South Wales; WA, Western Australia.

a Rate per 100 000 infants followed from 39 days to 1 year of age.

b Created by applying stabilized weights based on the inverse probability of treatment (IPT) methodology^19^ to adjust the demographic and risk characteristics in the on-time group to match the delayed group.

c Epidemiological measures were based on the weighted rates for the on-time (counterfactual) cohort.

### Sensitivity analysis

In the base case method for calculating the propensity score, if a covariate was missing it was assigned a separate category. In a sensitivity analysis we excluded subjects with at least one missing covariate.

## Aim 2-specific analysis methods

For each dose-specific analysis, infants meeting inclusion criteria were followed up from the date reaching the recommended age (or the 28th day since the previous dose if this date fell after the recommended age) for the dose under consideration, to the earliest of the following: first invalid DTP dose, death, fourth DTP dose or 1 year of age (i.e. the follow-up period).

The exposure cohorts for each dose-specific analysis were then defined as:

- on-time cohort—infants who received the dose under consideration and any subsequent doses on time;
- delayed cohort—infants who were ‘delayed’ in their receipt of the dose under consideration (despite being eligible to receive it on time and regardless of the timing of any subsequent doses); for the DTP1 analysis this also included infants who did not receive DTP1 within the follow-up period.

Stabilized IPT weights were calculated for each dose-specific cohort. Poisson regression models were then fitted to obtain weighted incidence rates of pertussis notification (see Supplementary Methods, for details).

## Calculation of epidemiological measures

The crude and weighted on-time ($I_{O}$) and delayed ($I_{D}$) incidence rates calculated for each aim were used to derive the rate of excess cases (i.e. absolute risk difference; ARD = $I_{D}-I_{O}$), the number of delayed infants needed to be vaccinated on time to prevent one case of pertussis $\left(\frac{100\;000}{I_{D}-I_{O}}\right)$ and the proportion of pertussis cases in the delayed cohort attributable to receiving at least one delayed dose (attributable fraction or AF = $\left[\left(1-\frac{I_{O}}{I_{D}}\right)\times 100\%\right]$).

## Results

### Study cohorts and weighting outcomes

Supplementary Figure S1 (available as Supplementary data at *IJE* online) describes the assembly of each analytical cohort. After applying the inclusion and exclusion criteria for the first aim, 820 001 births were included in the on-time cohort and 592 983 in the delayed cohort (1 412 984 infants in total accounting for 94.7% of the original linked cohort). After applying the additional dose-specific exclusion criteria, Aim 2 DTP1, 2 and 3 cohorts included 998 742, 1 140 723 and 1 295 150 infants, respectively. Compared with the on-time cohorts, the delayed cohorts had a different profile for most covariates examined (Table 2; Supplementary Table S1, available as Supplementary data at *IJE* online). In particular, the delayed cohorts were more likely to have a higher proportion of mothers with previous pregnancies, who smoked while pregnant, were Aboriginal or lived in remote regions. After applying the IPT weights, all standardized differences in covariate distributions between the delayed and on-time cohorts were below 0.15 and all except one covariate in each model was below 0.1 for the main analyses (Aims 1 and 2; Supplementary Table S2, available as Supplementary data at *IJE* online) and for the Aim 1 sub-analyses (Supplementary Table S3, available as Supplementary data at *IJE* online).

### Aim 1: overall excess burden of pertussis in the first year of life

Supplementary Table S4 (available as Supplementary data at *IJE* online) shows the number of subjects meeting the inclusion criteria for analysis of delayed and on-time cohorts and their respective number of notified cases in the Aim 1 main and subgroup analyses, with and without exclusion of children missing at least one covariate. Overall and for each subgroup, similar proportions of children were excluded due to missing values from the delayed and on-time cohorts, with the sub-analysis for Western Australia (WA) children only excluding the most children [due to higher proportions with a missing value for mother’s region of birth or socioeconomic/remoteness category than in New South Wales (NSW)].

The adjusted rate of pertussis for all eligible infants was estimated to be 53% higher in the delayed cohort compared with the counterfactual on-time cohort (Table 3). Overall, it was estimated that the excess burden in the first year of life was 85 (95% CI: 68–102) cases per 100 000 infants if, counter to the fact, all infants with at least one delayed dose had been vaccinated ontime, and 34% of cases in the delayed cohort were attributable to having at least one of the three primary doses after the on-time window.

**Figure 1:**
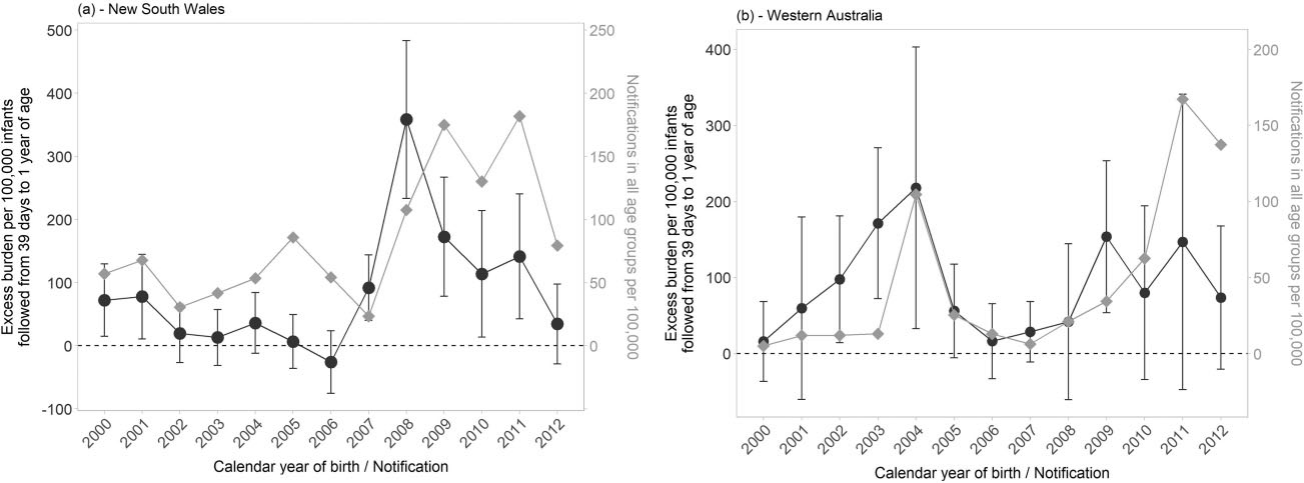
Birth cohort-specific excess burden of pertussis to 1 year of age (Aim 1 analysis) and number of pertussis notifications (all ages) by state and year

The rate of pertussis in the counterfactual on-time cohort varied across subgroups. However, the relative rates comparing the delayed and counterfactual on-time cohorts were similar (point estimates ranging 1.26 to 1.76), with all subgroups experiencing higher rates in the delayed infants. Aboriginal infants and infants born to families with at least three older siblings had the highest point estimates for excess rates of pertussis (163/100 000 infants for both). Overall, it was estimated that 1175 infants in the delayed cohort would need to be vaccinated on time to prevent one case. For Aboriginal infants and infants with three or more older siblings, the number needed to vaccinate reduced to 612 and 613, respectively. Most (88.6%) children in the delayed cohort had received at least one DTP dose by 1 year of age, and 29% of cases in this cohort could have been prevented if, counter to the fact, had they received all their vaccinations on time. The excess burden in infants was generally higher in years with a higher number of annual pertussis notifications (Figure 1).

Compared with the weighted analysis, the unweighted (crude) results, overall and in most subgroups examined, overestimated the number of excess cases (Table 3; Supplementary Table S5, available as Supplementary data at *IJE* online). The sensitivity analysis performed excluding the subjects with at least one missing value for covariates used in calculating IPT weights showed qualitatively similar results to the main analysis (Supplementary Table S6, available as Supplementary data at *IJE* online).

### Aim 2: dose-specific excess burden of pertussis in the first year of life

Table 4 shows the estimated dose-specific excess burden in the first year of life if the dose under consideration and all future doses (if any) in the delayed cohort were given on time. If all eligible infants with a delayed DTP1 had been vaccinated on time with all three primary doses, then an estimated 132 pertussis cases per 100 000 (49% of all cases in the DTP1 delayed cohort), could have been prevented. Whereas the estimated number of excess cases was lower for infants who had DTP1 on time but a delayed subsequent dose, the proportion of the excess cases remained considerable (37% for delayed DTP2 and 25% for delayed DTP3). Compared with the weighted rates, the unweighted (crude) rates for each dose were lower (Table 4).

**Table 4:** Unweighted and weighted rates of pertussis and epidemiological measures of the excess burden: Aim 2 analysis

Follow-up starting dose	Rate of pertussis in delayed cohort^a^ ($I_{D}$)	Unweighted rate of pertussis in on-time cohort^a^	Weighted rate of pertussis in on-time cohort^a^^,^^b^ ($I_{o}$)	Relative rate^c^$\left(\frac{I_{D}}{I_{O}}\right)$	Excess cases^a^^,^^c^$\left(I_{D}-I_{O}\right)$	Number needed to vaccinate on time to prevent one case of pertussis^c^$\left(\frac{100\;000}{I_{D}-I_{O}}\right)$	Attributable fraction (%)^c^$\left[\left(1-\frac{I_{o}}{I_{D}}\right)\times 100\%\right]$
DTP1	273 (248, 297)	120 (113, 128)	140 (128, 153)	1.9 (1.7, 2.2)	132 (105, 160)	755 (599, 911)	49 (42, 55)
DTP2	136 (123, 149)	75 (69, 81)	86 (78, 95)	1.6 (1.4, 1.8)	50 (35, 65)	2013 (1402, 2624)	37 (28, 45)
DTP3	78 (70, 86)	56 (51, 61)	59 (53, 64)	1.3 (1.1, 1.5)	19 (9, 29)	5184 (2491, 7877)	25 (14, 36)

DTP, diphtheria-tetanus-pertussis vaccine.

a Rate per 100 000 infants followed from recommended age of vaccination to 1 year of age.

b Created by applying stabilized weights based on the inverse probability of treatment (IPT) methodology^19^ to adjust the demographic and risk characteristics in the on-time cohort to match the delayed cohort.

c Epidemiological measures were based on the weighted rates for the on-time (counterfactual) cohort.

## Discussion

To our knowledge this is the first study to estimate the excess burden of pertussis that might have been prevented by improving vaccination timeliness, using a population cohort approach with individual-level data. Using a potential outcomes framework we estimated that on average 85 cases/100 000 infants with at least one delayed dose in their first year of life could have been prevented during the study period if, counter to the fact, they had been vaccinated on time. This is significant given that 42% of infants in our study had at least one delayed dose and that morbidity in infants is considerable (>30% of cases in <1-year-olds are hospitalized^17^ and they account for >85% of all pertussis-related intensive care unit admissions).^21^ Rates of pertussis in the delayed cohort were 53% higher overall than in the counterfactual on-time control cohort. Most (89%) infants in the delayed cohort received at least one dose, and an estimated 29% of pertussis cases could have been prevented in this cohort if all their doses had been given on time, suggesting that targeted programmes to improve timeliness in partially vaccinated infants would be beneficial. Whereas the relative increase was similar across the different subpopulations examined, the absolute risk differences varied due to differences in rates of pertussis in the counterfactual on-time cohorts; the greatest impact of improving timeliness was in epidemic years, infants with three or more older siblings and Aboriginal infants (although confidence intervals overlapped with those of other subgroups).

Our study builds on previous findings using the same linked data. Our study suggests that subgroups already identified as having low on-time coverage^5^^,^^6^ would benefit significantly from efforts to improve timeliness. Infants with three or more older siblings and Aboriginal infants have been reported to have among the lowest levels of on-time coverage^5^ and a higher proportion with long delays,^6^ and in our study they also had a higher rate of pertussis than other subgroups examined. These factors help explain why they had the highest excess rate of pertussis.

We found improving Dose 1 timeliness would prevent the most cases in the delayed cohort, consistent with the highest burden of pertussis being among 1–3-month olds.^3^ Getting Dose 1 on time has previously been reported to halve the risk of delayed subsequent doses^6^ and thus would have significant flow-on effects for disease prevention.

Few studies have examined the potential benefit of improving timeliness, with those conducted to date (in the USA, Argentina and Flanders) reliant on modelling approaches using unlinked data.^10–13^ All found that improving timeliness would reduce the incidence of pertussis. However, the estimated impact varied considerably, with rate differences an order of magnitude lower, in most instances, than found in our study (1.7 to 6.6 cases/100 000 infants <1 year of age; 9–20% rate reduction).^10^^,^^11^^,^^13^ In contrast, a study in Sweden reported a rate difference of 129/100 000 (41% rate reduction) for DTP1 and a 28% reduction overall in cases occurring up to 2 years of age, which are more similar to our findings.^12^ Differences between studies are mainly due to substantially lower reported rates of pertussis in the USA, Argentina and Flanders than in Sweden and Australia, but may also be related to differences in the data sources used, vaccination schedules, degree of delay in vaccination and analytical methods (including follow-up time).

The key strengths of our study are that we used individual-level data from a large population-based cohort and a potential outcomes framework by creating counterfactual on-time cohorts. Balancing covariate distributions of the on-time cohort to match those of the delayed cohort was critical because those demographic groups at higher risk of vaccine delay were also at a higher risk of getting pertussis^6^; this led to an overestimate of the impact of improving vaccination timeliness in the Aim 1 unweighted analysis. We assumed that, after weighting, any pertussis rate differences between the delayed and counterfactual on-time cohorts were associated with vaccination delay. However, it must be noted that infants were classified as delayed based on at least one dose being delayed, but the outcome (pertussis) could have occurred at any time during follow-up to 1 year of age (i.e. may have occurred after receipt of an on-time dose). In addition, the propensity weighting process may not have controlled for all confounding. Therefore, although a potential outcomes approach enabled robust assessment of the excess burden of pertussis, we must be cautious about interpreting the effects as causal. Our methods did not enable estimation of any population-level impact (herd effects) of improving vaccination timeliness which, even if minimal, would mean the benefit is even greater than we have estimated. Additional modelling may also assist policy makers to determine the best timing of any catch-up campaigns with respect to potential seasonal and epidemic caseloads. The smaller size of some subgroups meant that some sub-analyses were underpowered. Finally, our results may not be generalizable to other settings, due to differences in pertussis epidemiology, degree of delay in vaccination and vaccine scheduling (including maternal immunization, which was introduced in Australia after this study). We chose to dichotomize the on-time and delayed categories according to those used by policy makers. This simplifies the propensity matching of the on-time to the delayed groups, and makes the results easier for policy-relevant communication. The methodology presented here can therefore easily be implemented in other countries with individual-level data to obtain accurate estimates of their excess burden.

## Conclusion

In conclusion, our study provides robust quantitative evidence that improving DTP vaccine timeliness reduces the burden of infant pertussis and identifies key population subgroups who would benefit most from targeted interventions to reduce delays. These findings can inform cost-benefit analyses of targeted programmes and public health messaging to reduce delays. The study also highlights that subpopulations with delayed vaccination have a greater underlying risk of pertussis than infants vaccinated on time, an important consideration for future studies evaluating the benefit of improving timeliness.

## Ethics approval

Ethics approval was obtained from the Australian Institute of Health and Welfare, the NSW Population & Health Services Research Ethics Committee, Department of Health WA Human Research Ethics Committee, the WA Aboriginal Health Ethics Committee, the NSW Aboriginal Health and Medical Research Council Ethics Committee and the Australian Government Department of Health and Ageing Departmental Ethics Committee.

## Supplementary Material

dyac175_Supplementary_Data

## Data Availability

The data underlying this article cannot be shared publicly due to ethical and privacy constraints. However, the programming code is available upon request.
