# Supplementary material for: Estimating the excess burden of pertussis disease in Australia within the first year of life, that might have been prevented through timely vaccination
Source: Int J Epidemiol. 2022 Sep 13;52(1):250–9. doi: 10.1093/ije/dyac175 (PMC9908038; doi:10.1093/ije/dyac175)
Supplement: dyac175_Supplementary_Data [file dyac175_supplementary_data.docx]

# Supplementary Methods

**Additional details regarding exclusion and inclusion criteria for Aim 2**

For the dose-specific analysis (Aim 2), we had the following additional exclusion criteria:

1. Notified with pertussis before the recommended age (or before the 28^th^ day since the previous dose if this date fell after the recommended age) of the dose under consideration,
2. Died before the oldest age that the dose under consideration can be received ‘on-time’,
3. Previous doses (if any) had been received such that the dose under consideration could not be received within the ‘on-time’ window while allowing 28 days between successive doses (i.e., the infant had received a prior dose <13 days before the due date of the dose under consideration such that a minimum of 28 days between doses and enough time to receive the dose ≤15 days after the due date could not be met),
4. Received the dose under consideration before the ‘on-time’ window, or
5. Received the dose under consideration within the ‘on-time’ window but any subsequent doses recommended in the first year of life were ‘delayed’.

**Propensity weighting of ‘on-time’ cohorts**

The weighting process used here enabled application of a potential outcomes framework to assess the ‘average treatment effect in the untreated’ (ATU), i.e., how the rate of pertussis, on average, would differ in the ‘delayed’ cohort if, counter to the fact, they had been vaccinated on time.^1^

Logistic regression was used to estimate the propensity score (p) of being ‘delayed’ for all infants based on their gestational age group, parity, Aboriginal status, year of birth, season of birth, Socio-economic Indices for Areas (SEIFA) quintile (measured using the Index of Relative Socio-economic Disadvantage; IRSD),^2^ mother’s age group, smoking status during pregnancy, remoteness category (measured using the Accessibility/Remoteness Index of Australia; ARIA),^3^ mother’s region of birth, and jurisdictional state of birth registration (see Table S1 for variable categories). SEIFA and ARIA categories were based on mother’s residential postcode at the time of birth. The infants in the ‘delayed’ cohort were given the weight of 1 and the infants in the ‘on-time’ (control) cohort were given a weight of $\left( \frac{\text{proportion of subjects in the 'on-time' cohort}}{\text{proportion of subjects in the 'delayed' cohort}} \right)\times\left( \frac{p}{1-p} \right)$.^4^ This weighting scheme was expected to make the weighted distribution of covariates in the ‘on-time’ cohort similar to the ‘delayed’ cohort while preserving the sample sizes in each exposure cohort. Standardised differences^4^ in the covariates between the two exposure cohorts were used to assess the degree of agreement of distributions before and after stabilised IPT weighting. Even though there is no fixed cut-off, standardised differences <0.1 were considered a satisfactory level of agreement between the covariate distributions.^5^

**Calculation of incidence rates of pertussis notification**

Poisson regression models with robust error variances were fitted without (Aim 1 only) and with stabilised IPT weights to obtain crude and weighted incidence rates of pertussis notification, respectively.

Aim 1 rates:

Per 326 x 100,000 person-days; equivalent to the person time of 100,000 infants followed up from 39 days to 1 year of age) at any time during the follow up period in the two exposure cohorts (‘on-time’ ($I_{O}$) and ‘delayed’ ($I_{D}$).

Aim 2 rates:

For Diphtheria-Tetanus-Pertussis vaccine dose 1 (DTP1): per 304 x 100,000 person-days, equivalent to the person time of 100,000 infants followed up from 61 days to 1 year of age;

For DTP2: per 243 x 100,000 person-days, equivalent to the person time of 100,000 infants followed up from 122 days to 1 year of age; and

For DTP3: per 182 x 100,000 person-days, equivalent to the person time of 100,000 infants followed up from 183 days to 1 year of age.

# Supplementary Results

**Supplementary Figure S1 - Assembly of the study cohorts used in Aims 1 and 2**


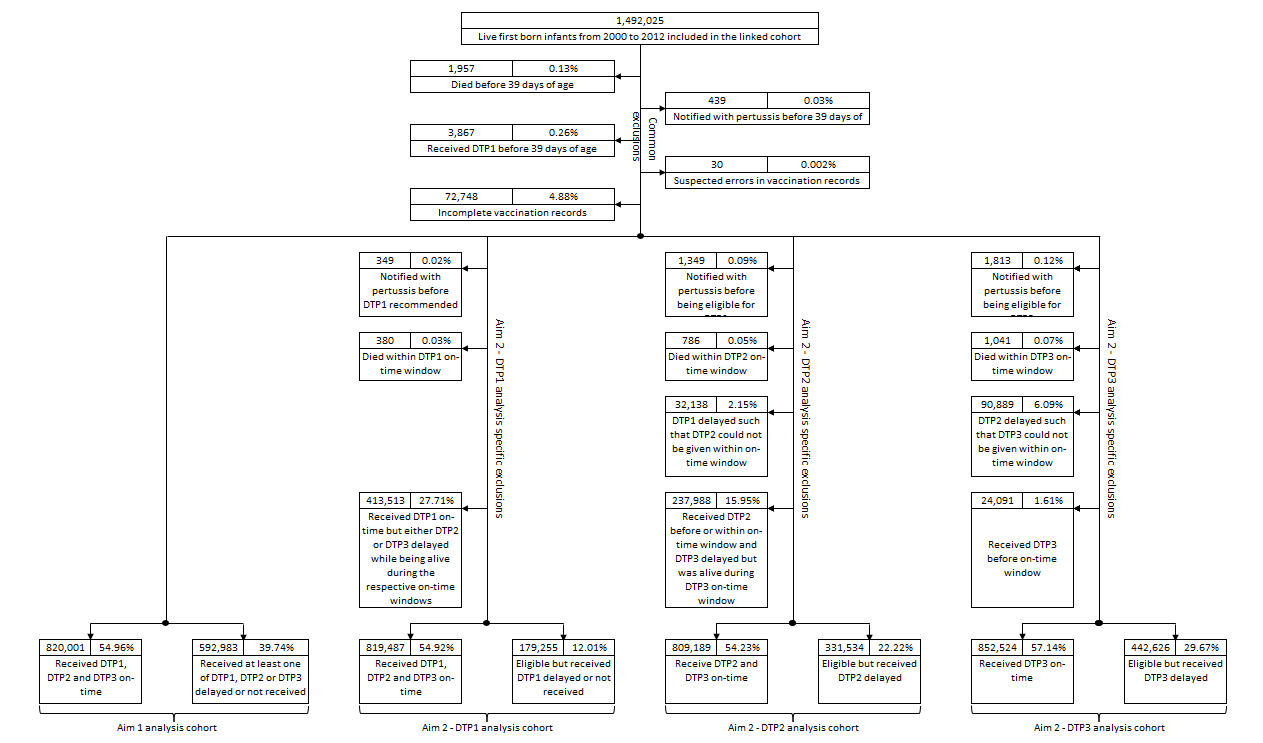


DTP, Diphtheria-Tetanus-Pertussis vaccine

**Supplementary Table S1 - Characteristics of the study cohorts**

|  | **Aim 1** | |  | **Aim 2 - DTP1 analysis** | |  | **Aim 2 - DTP2 analysis** | |  | **Aim 2 - DTP3 analysis** | |
| --- | --- | --- | --- | --- | --- | --- | --- | --- | --- | --- | --- |
|  | **‘On-time’**  **(n = 820,001)** | **‘Delayed’**  **(n = 592,983)** |  | **‘On-time’**  **(n = 819,487)** | **‘Delayed’**  **(n = 179,255)** |  | **‘On-time’**  **(n = 809,189)** | **‘Delayed’**  **(n = 331,534)** |  | **‘On-time’**  **(n = 852,524)** | **‘Delayed’**  **(n = 442,626)** |
| Gestational age | | | | | | | | | | | |
| <=28 weeks | 0.16% | 0.37% |  | 0.16% | 0.58% |  | 0.16% | 0.42% |  | 0.16% | 0.32% |
| 29-32 weeks | 0.60% | 0.89% |  | 0.60% | 1.22% |  | 0.61% | 0.94% |  | 0.61% | 0.83% |
| 33-36 weeks | 4.96% | 5.51% |  | 4.96% | 6.34% |  | 4.98% | 5.54% |  | 4.97% | 5.30% |
| 37-40 weeks | 78.25% | 77.94% |  | 78.26% | 75.79% |  | 78.23% | 77.66% |  | 78.30% | 78.18% |
| >40 weeks | 16.02% | 15.27% |  | 16.02% | 16.04% |  | 16.01% | 15.41% |  | 15.95% | 15.35% |
| Missing | 0.01% | 0.02% |  | 0.01% | 0.03% |  | 0.01% | 0.02% |  | 0.01% | 0.02% |
| Parity | | | | | | | | | | | |
| 0 | 48.03% | 27.98% |  | 48.04% | 27.32% |  | 47.70% | 26.60% |  | 46.70% | 29.75% |
| 1-2 | 49.04% | 63.87% |  | 49.04% | 61.28% |  | 49.35% | 64.61% |  | 50.21% | 63.21% |
| 3+ | 2.84% | 8.04% |  | 2.83% | 11.28% |  | 2.86% | 8.68% |  | 2.99% | 6.93% |
| Missing | 0.09% | 0.11% |  | 0.09% | 0.11% |  | 0.09% | 0.11% |  | 0.10% | 0.11% |
| Aboriginal status | | | | | | | | | | | |
| Non-Aboriginal | 96.76% | 92.90% |  | 96.76% | 89.99% |  | 96.75% | 92.38% |  | 96.63% | 94.03% |
| Aboriginal | 3.24% | 7.10% |  | 3.24% | 10.01% |  | 3.25% | 7.61% |  | 3.37% | 5.97% |
| Missing | 0.00% | 0.00% |  | 0.00% | 0.00% |  | 0.00% | 0.00% |  | 0.00% | 0.00% |
| Year of birth | | | | | | | | | | | |
| 2000 | 7.00% | 7.71% |  | 7.00% | 8.77% |  | 7.07% | 7.94% |  | 7.00% | 7.81% |
| 2001 | 7.06% | 7.41% |  | 7.06% | 8.46% |  | 7.15% | 7.55% |  | 7.08% | 7.45% |
| 2002 | 7.04% | 7.41% |  | 7.05% | 8.31% |  | 7.14% | 7.51% |  | 7.11% | 7.41% |
| 2003 | 6.85% | 7.76% |  | 6.85% | 8.65% |  | 6.96% | 7.85% |  | 6.95% | 7.61% |
| 2004 | 6.85% | 7.68% |  | 6.85% | 8.41% |  | 6.95% | 7.73% |  | 6.92% | 7.58% |
| 2005 | 7.29% | 7.89% |  | 7.29% | 8.51% |  | 7.39% | 7.90% |  | 7.34% | 7.75% |
| 2006 | 7.81% | 7.79% |  | 7.81% | 8.13% |  | 7.92% | 7.62% |  | 7.85% | 7.75% |
| 2007 | 8.10% | 8.12% |  | 8.10% | 8.14% |  | 8.21% | 7.91% |  | 8.16% | 8.06% |
| 2008 | 8.34% | 7.85% |  | 8.34% | 8.32% |  | 8.48% | 7.75% |  | 8.44% | 7.64% |
| 2009 | 8.43% | 7.62% |  | 8.43% | 6.52% |  | 8.23% | 7.46% |  | 8.30% | 7.63% |
| 2010 | 8.38% | 7.72% |  | 8.38% | 6.51% |  | 8.16% | 7.69% |  | 8.29% | 7.73% |
| 2011 | 8.54% | 7.61% |  | 8.54% | 5.79% |  | 8.24% | 7.56% |  | 8.36% | 7.84% |
| 2012 | 8.30% | 7.43% |  | 8.30% | 5.49% |  | 8.10% | 7.54% |  | 8.21% | 7.74% |
| Season of birth | | | | | | | | | | | |
| Spring | 25.75% | 24.53% |  | 25.75% | 25.28% |  | 25.83% | 24.20% |  | 25.80% | 24.58% |
| Summer | 24.89% | 25.80% |  | 24.89% | 25.08% |  | 24.84% | 25.93% |  | 24.94% | 25.79% |
| Autumn | 25.16% | 26.00% |  | 25.16% | 25.98% |  | 25.14% | 26.31% |  | 25.22% | 25.90% |
| Winter | 24.21% | 23.67% |  | 24.21% | 23.67% |  | 24.19% | 23.56% |  | 24.04% | 23.73% |
| SEIFA quintile^a^ | | | | | | | | | | | |
| 91-100% | 9.23% | 8.72% |  | 9.23% | 7.74% |  | 9.18% | 8.38% |  | 9.23% | 9.14% |
| 76-90% | 15.78% | 14.06% |  | 15.78% | 12.30% |  | 15.73% | 13.54% |  | 15.72% | 14.64% |
| 26-75% | 48.43% | 46.18% |  | 48.44% | 44.45% |  | 48.44% | 45.92% |  | 48.30% | 46.58% |
| 11-25% | 14.74% | 15.86% |  | 14.74% | 17.14% |  | 14.76% | 16.29% |  | 14.78% | 15.44% |
| 0-10% | 9.29% | 11.89% |  | 9.28% | 14.42% |  | 9.34% | 12.53% |  | 9.45% | 10.92% |
| Missing | 2.53% | 3.27% |  | 2.53% | 3.95% |  | 2.54% | 3.35% |  | 2.52% | 3.28% |
| Mother's age | | | | | | | | | | | |
| >=35 years | 20.64% | 21.85% |  | 20.65% | 21.79% |  | 20.63% | 21.84% |  | 20.74% | 22.19% |
| 30-34 years | 33.81% | 31.36% |  | 33.81% | 28.62% |  | 33.78% | 30.57% |  | 33.74% | 32.07% |
| 25-29 years | 28.95% | 26.61% |  | 28.96% | 26.18% |  | 28.95% | 26.45% |  | 28.77% | 26.70% |
| 20-24 years | 13.28% | 15.42% |  | 13.28% | 17.41% |  | 13.32% | 16.03% |  | 13.37% | 14.64% |
| <20 years | 3.32% | 4.76% |  | 3.31% | 6.00% |  | 3.33% | 5.11% |  | 3.38% | 4.40% |
| Smoking status during pregnancy | | | | | | | | | | | |
| No | 89.31% | 82.00% |  | 89.33% | 77.58% |  | 89.29% | 81.07% |  | 89.12% | 83.81% |
| Yes | 10.47% | 17.82% |  | 10.46% | 22.22% |  | 10.50% | 18.75% |  | 10.67% | 16.01% |
| Missing | 0.21% | 0.19% |  | 0.21% | 0.20% |  | 0.21% | 0.18% |  | 0.21% | 0.18% |
| Remoteness category^b^ | | | | | | | | | | | |
| Major cities | 75.67% | 71.90% |  | 75.68% | 70.25% |  | 75.70% | 71.61% |  | 75.77% | 72.30% |
| Inner regional | 14.80% | 16.04% |  | 14.80% | 16.44% |  | 14.76% | 16.14% |  | 14.74% | 15.90% |
| Outer regional | 5.63% | 6.78% |  | 5.63% | 6.99% |  | 5.62% | 6.87% |  | 5.60% | 6.65% |
| Remote | 1.56% | 2.23% |  | 1.56% | 2.67% |  | 1.56% | 2.28% |  | 1.56% | 2.08% |
| Missing | 2.34% | 3.05% |  | 2.34% | 3.66% |  | 2.35% | 3.10% |  | 2.33% | 3.07% |
| Mother's region of birth | | | | | | | | | | | |
| Australia | 67.52% | 68.86% |  | 67.52% | 65.11% |  | 67.57% | 67.62% |  | 67.51% | 68.30% |
| Oceania | 3.02% | 5.04% |  | 3.02% | 6.42% |  | 3.05% | 5.36% |  | 3.15% | 4.58% |
| Europe | 6.05% | 6.34% |  | 6.05% | 6.19% |  | 5.99% | 6.26% |  | 6.02% | 6.66% |
| Middle East and Africa | 5.37% | 5.14% |  | 5.37% | 5.48% |  | 5.41% | 5.33% |  | 5.46% | 5.19% |
| Asia | 13.69% | 9.62% |  | 13.70% | 11.44% |  | 13.63% | 10.29% |  | 13.56% | 10.14% |
| America | 1.36% | 1.62% |  | 1.36% | 2.12% |  | 1.35% | 1.77% |  | 1.36% | 1.71% |
| Missing | 2.99% | 3.39% |  | 2.99% | 3.23% |  | 3.00% | 3.37% |  | 2.94% | 3.43% |
| State of birth registration | | | | | | | | | | | |
| Western Australia | 22.24% | 26.75% |  | 22.24% | 27.39% |  | 22.36% | 26.41% |  | 22.11% | 26.61% |
| New South Wales | 77.76% | 73.25% |  | 77.76% | 72.61% |  | 77.64% | 73.59% |  | 77.89% | 73.39% |

DTP, Diphtheria-Tetanus-Pertussis vaccine

^a^ SEIFA, socio-economic indexes for areas. Measured using the Index of Relative Socio-economic Disadvantage (IRSD),^2^

^b^ Measured using the Accessibility/Remoteness Index of Australia (ARIA)^3^

**Supplementary Table S2 - Comparison of unweighted and weighted standardised differences between the two exposure groups in the cohorts used for Aims 1 and 2**

|  | **Aim 1** | |  | **Aim 2 - DTP1 analysis** | |  | **Aim 2 - DTP2 analysis** | |  | **Aim 2 - DTP3 analysis** | |
| --- | --- | --- | --- | --- | --- | --- | --- | --- | --- | --- | --- |
| **Covariate** | **Unweighted** | **Weighted***^a^* |  | **Unweighted** | **Weighted***^a^* |  | **Unweighted** | **Weighted***^a^* |  | **Unweighted** | **Weighted***^a^* |
| Gestational age | 0.050 | 0.148 |  | 0.150 | 0.000 |  | 0.050 | 0.148 |  | 0.144 | 0.000 |
| Parity | 0.447 | 0.000 |  | 0.522 | 0.143 |  | 0.462 | 0.000 |  | 0.377 | 0.000 |
| Aboriginal status | 0.184 | 0.000 |  | 0.287 | 0.000 |  | 0.221 | 0.000 |  | 0.145 | 0.000 |
| Year of birth | 0.136 | 0.000 |  | 0.239 | 0.055 |  | 0.208 | 0.097 |  | 0.160 | 0.000 |
| Season of birth | 0.040 | 0.000 |  | 0.028 | 0.000 |  | 0.049 | 0.000 |  | 0.040 | 0.000 |
| SEIFA quintile^b^ | 0.113 | 0.000 |  | 0.226 | 0.050 |  | 0.141 | 0.061 |  | 0.070 | 0.000 |
| Mother's age | 0.133 | 0.047 |  | 0.207 | 0.044 |  | 0.151 | 0.046 |  | 0.094 | 0.000 |
| Smoking status during pregnancy | 0.270 | 0.000 |  | 0.359 | 0.000 |  | 0.292 | 0.000 |  | 0.147 | 0.000 |
| Remoteness category^c^ | 0.157 | 0.000 |  | 0.218 | 0.000 |  | 0.157 | 0.055 |  | 0.157 | 0.000 |
| Mother's region of birth | 0.174 | 0.055 |  | 0.214 | 0.071 |  | 0.182 | 0.033 |  | 0.178 | 0.000 |
| State of birth registration | 0.105 | -0.004 |  | 0.120 | -0.004 |  | 0.094 | -0.004 |  | 0.105 | -0.004 |

^a^ Absolute standardised difference < 0.1 generally indicates satisfactory level of agreement between the covariate distributions.

^b^ SEIFA, socio-economic indexes for areas. Measured using the Index of Relative Socio-economic Disadvantage (IRSD),^2^

^c^ Measured using the Accessibility/Remoteness Index of Australia (ARIA)^3^

***Supplementary Table S3 – Standardised differences in covariate distributions between the two exposure groups before (U) and after (W) IPT weighting (all infants meeting study inclusion criteria)^a^ for each sub-group analysis***

| **Covariate** | **All groups** | | **Birth registered in NSW** | | **Birth registered in WA** | | **At least 1 dose in the 1^st^ year of life** | | **Non-Indigenous** | | **Indigenous** | | **Gestational age <=36 weeks** | | **Parity >=3** | | **Mother born in Oceania excluding Australia** | | **Mother aged < 20 years** | | **Mother smoked during pregnancy** | |
| --- | --- | --- | --- | --- | --- | --- | --- | --- | --- | --- | --- | --- | --- | --- | --- | --- | --- | --- | --- | --- | --- | --- |
|  | **U** | **W**^b^ | **U** | **W**^b^ | **U** | **W**^b^ | **U** | **W**^b^ | **U** | **W**^b^ | **U** | **W**^b^ | **U** | **W**^b^ | **U** | **W**^b^ | **U** | **W**^b^ | **U** | **W**^b^ | **U** | **W**^b^ |
| Gestational age group | 0.05 | 0.15 | 0.00 | 0.00 | 0.15 | 0.00 | 0.05 | 0.00 | 0.14 | 0.00 | 0.13 | 0.00 | 0.14 | 0.04 | 0.21 | 0.00 | 0.15 | 0.00 | 0.18 | 0.00 | 0.18 | 0.00 |
| Parity group | 0.45 | 0.00 | 0.46 | 0.00 | 0.46 | 0.00 | 0.47 | 0.00 | 0.44 | 0.00 | 0.49 | 0.00 | 0.48 | 0.00 | - | - | 0.51 | 0.00 | 0.30 | 0.00 | 0.52 | 0.14 |
| Aboriginal status | 0.18 | 0.00 | 0.15 | 0.00 | 0.26 | 0.00 | 0.17 | 0.00 | - | - | - | - | 0.25 | 0.00 | 0.27 | 0.00 | 0.08 | 0.00 | 0.24 | 0.00 | 0.22 | 0.00 |
| Year of birth | 0.14 | 0.00 | 0.18 | 0.06 | 0.09 | 0.00 | 0.14 | 0.00 | 0.16 | 0.00 | 0.16 | 0.05 | 0.10 | 0.00 | 0.10 | 0.00 | 0.16 | 0.05 | 0.07 | 0.06 | 0.10 | 0.05 |
| Season of birth | 0.04 | 0.00 | 0.04 | 0.00 | 0.05 | 0.00 | 0.05 | 0.03 | 0.04 | 0.00 | 0.04 | 0.00 | 0.03 | 0.00 | 0.05 | 0.00 | 0.04 | 0.00 | 0.04 | 0.03 | 0.05 | 0.00 |
| SEIFA Quintile^c^ | 0.11 | 0.00 | 0.18 | 0.09 | 0.13 | 0.00 | 0.11 | 0.00 | 0.08 | 0.00 | 0.19 | 0.00 | 0.19 | 0.00 | 0.12 | 0.05 | 0.23 | 0.00 | 0.14 | 0.11 | 0.17 | 0.06 |
| Mother’s age group (years) | 0.13 | 0.05 | 0.09 | 0.03 | 0.11 | 0.03 | 0.09 | 0.05 | 0.07 | 0.06 | 0.03 | 0.06 | 0.18 | 0.04 | 0.10 | 0.00 | 0.14 | 0.03 | - | - | 0.05 | 0.03 |
| Smoking status during pregnancy | 0.27 | 0.00 | 0.21 | 0.00 | 0.21 | 0.00 | 0.27 | 0.00 | 0.15 | 0.00 | 0.28 | 0.00 | 0.38 | 0.00 | 0.30 | 0.02 | 0.21 | 0.00 | 0.17 | 0.00 | - | - |
| Remoteness category^d^ | 0.16 | 0.00 | 0.16 | 0.00 | 0.10 | 0.00 | 0.16 | 0.00 | 0.08 | 0.00 | 0.15 | 0.06 | 0.13 | 0.00 | 0.08 | 0.00 | 0.15 | 0.00 | 0.12 | 0.00 | 0.09 | 0.00 |
| Mother’s region of birth | 0.17 | 0.05 | 0.23 | 0.15 | 0.14 | 0.07 | 0.19 | 0.00 | 0.18 | 0.03 | 0.23 | 0.04 | 0.17 | 0.00 | 0.32 | 0.00 | - | - | 0.16 | 0.00 | 0.13 | 0.00 |
| State of birth registration | 0.10 | 0.00 | - | - | - | - | 0.11 | 0.00 | 0.09 | -0.01 | 0.30 | -0.02 | 0.14 | 0.00 | -0.02 | -0.01 | -0.01 | -0.01 | 0.15 | -0.01 | 0.10 | 0.00 |

^a^ Infants who have at least one covariate out of those used to calculate propensity score recorded as missing do not get a valid propensity score. In the base case, we categorised all missing records under each covariate as a separate category/class specific to that covariate. As a sensitivity analysis (see Supplementary Table S6) we excluded all infants who have at least one covariate out of those used to calculate propensity score recorded as missing (This sample corresponds to the numbers mentioned under “Sample excluding missing data” in Supplementary Table S4).

^b^ Absolute standardised difference < 0.1 generally indicates satisfactory level of agreement between the covariate distributions.

^c^ Measured using the Index of Relative Socio-economic Disadvantage (IRSD),^2^

^d^ Measured using the Accessibility/Remoteness Index of Australia (ARIA)^3^

**Supplementary Table S4 - Sample size comparison for Aim 1 before and after excluding subjects with** **at least one missing covariate used to calculate propensity scores**

| **Population sub-group** | **All infants meeting inclusion criteria** | | | | **Sample excluding missing data** | | | | **% excluded from Delayed** | **% excluded from  On-time** |
| --- | --- | --- | --- | --- | --- | --- | --- | --- | --- | --- |
|  | **Delayed** | | **On-time** | | **Delayed** | | **On-time** | |  |  |
|  | **Number of subjects** | **Pertussis cases** | **Number of subjects** | **Pertussis cases** | **Number of subjects** | **Pertussis cases** | **Number of subjects** | **Pertussis cases** |  |  |
| All groups | 592,983 | 1,461 | 820,001 | 1,083 | 553,583 | 1,406 | 774,275 | 1,046 | 7% | 6% |
| Birth registered in New South Wales | 434,368 | 1,137 | 637,599 | 921 | 425,256 | 1,128 | 626,391 | 910 | 2% | 2% |
| Birth registered in Western Australia | 158,615 | 324 | 182,402 | 162 | 128,327 | 278 | 147,884 | 136 | 19% | 19% |
| Non-Aboriginal | 550,885 | 1,260 | 793,421 | 1,002 | 515,557 | 1,219 | 749,504 | 972 | 6% | 6% |
| Aboriginal | 42,098 | 201 | 26,580 | 81 | 38,029 | 187 | 24,771 | 74 | 10% | 7% |
| Gestational age <=36 weeks | 40,218 | 134 | 47,031 | 87 | 37,033 | 127 | 43,859 | 84 | 8% | 7% |
| Parity >=3 | 47,657 | 231 | 23,254 | 73 | 42,782 | 221 | 20,956 | 66 | 10% | 10% |
| Mother born in Oceania excluding Australia | 29,858 | 85 | 24,800 | 37 | 28,898 | 83 | 23,884 | 37 | 3% | 4% |
| Mother aged < 20 years | 28,202 | 98 | 27,185 | 60 | 25,878 | 91 | 25,368 | 57 | 8% | 7% |
| Mother smoked during pregnancy | 105,662 | 332 | 85,887 | 154 | 97,370 | 320 | 79,401 | 145 | 8% | 8% |
| At least 1 dose within the 1st year of life | 525,272 | 1,197 | 820,001 | 1,083 | 490,953 | 1,153 | 774,275 | 1,046 | 7% | 6% |

**Supplementary Table S5 - Crude rates of pertussis and epidemiologic measures of the excess burden (all infants meeting Aim 1 study inclusion criteria)**

| **Population sub-group** | **Rate of pertussis in 'delayed' cohort^a^**  **(**$\boldsymbol{I}_{\boldsymbol{D}}$**)** | **Rate of pertussis in unweighted ‘on-time’ cohort^a^**  **(**$\boldsymbol{I}_{\boldsymbol{o}}$**)** | **Relative rate^a^**  $\left( \frac{\boldsymbol{I}_{\boldsymbol{D}}}{\boldsymbol{I}_{\boldsymbol{O}}} \right)$ | **Excess Cases^a^**  $\boldsymbol{(}\boldsymbol{I}_{\boldsymbol{D}}\boldsymbol{-}\boldsymbol{I}_{\boldsymbol{O}}\boldsymbol{)}$ | **Number needed to vaccinate ‘on-time’ to prevent one case of pertussis**  $\left( \frac{\boldsymbol{100,000}}{\boldsymbol{I}_{\boldsymbol{D}}\boldsymbol{-}\boldsymbol{I}_{\boldsymbol{O}}} \right)$ | **Attributable Fraction (%)**  $\left[ \left( \boldsymbol{1-}\frac{\boldsymbol{I}_{\boldsymbol{o}}}{\boldsymbol{I}_{\boldsymbol{D}}} \right)\boldsymbol{\times100\%} \right]$ |
| --- | --- | --- | --- | --- | --- | --- |
| All groups | 247 (234, 259) | 137 (129, 145) | 1.80 (1.66, 1.94) | 110 (95, 125) | 911 (786, 1036) | 44 (40, 49) |
| Birth registered in New South Wales | 262 (247, 277) | 150 (140, 160) | 1.75 (1.60, 1.90) | 112 (94, 130) | 891 (748, 1034) | 43 (38, 48) |
| Birth registered in Western Australia | 205 (182, 227) | 92 (78, 106) | 2.22 (1.80, 2.64) | 113 (86, 139) | 888 (680, 1097) | 55 (47, 63) |
| Non-Aboriginal | 229 (216, 242) | 131 (123, 139) | 1.75 (1.60, 1.89) | 98 (83, 113) | 1019 (863, 1175) | 43 (38, 48) |
| Aboriginal | 479 (413, 545) | 321 (251, 391) | 1.49 (1.11, 1.87) | 157 (61, 253) | 635 (248, 1023) | 33 (16, 50) |
| gestational age <=36 weeks | 335 (279, 392) | 193 (152, 233) | 1.74 (1.27, 2.21) | 142 (73, 212) | 703 (359, 1047) | 42 (27, 58) |
| Parity >=3 | 486 (423, 548) | 327 (252, 402) | 1.48 (1.09, 1.87) | 159 (61, 256) | 631 (243, 1019) | 33 (15, 50) |
| Mother born in Oceania excluding Australia | 285 (225, 346) | 155 (105, 205) | 1.84 (1.13, 2.55) | 130 (52, 208) | 769 (305, 1233) | 46 (25, 67) |
| Mother aged < 20 years | 348 (279, 417) | 231 (173, 290) | 1.51 (1.02, 1.99) | 117 (27, 207) | 856 (194, 1517) | 34 (12, 55) |
| Mother smoked during pregnancy | 315 (281, 349) | 188 (158, 217) | 1.68 (1.36, 2.00) | 127 (82, 172) | 787 (509, 1065) | 40 (29, 52) |
| At least 1 dose within the 1st year of life | 228 (215, 241) | 137 (129, 145) | 1.67 (1.53, 1.80) | 91 (76, 107) | 1096 (912, 1279) | 40 (35, 45) |

^a^ Rate per 100,00 infants followed from 39 days to 1 year of age.

**Supplementary Table S6 - Weighted rates of pertussis and epidemiologic measures of the excess burden (sample excluding missing data)**

| **Population sub-group** | **Rate of pertussis in 'delayed' cohort^a^**  **(**$\boldsymbol{I}_{\boldsymbol{D}}$**)** | **Rate of pertussis in counterfactual ‘on-time’ cohort^a,b^**  **(**$\boldsymbol{I}_{\boldsymbol{o}}$**)** | **Relative rate**  $\left( \frac{\boldsymbol{I}_{\boldsymbol{D}}}{\boldsymbol{I}_{\boldsymbol{O}}} \right)$ | **Excess cases^a^**  $\boldsymbol{(}\boldsymbol{I}_{\boldsymbol{D}}\boldsymbol{-}\boldsymbol{I}_{\boldsymbol{O}}\boldsymbol{)}$ | **Number needed to vaccinate ‘on-time’ to prevent one case of pertussis**  $\left( \frac{\boldsymbol{100,000}}{\boldsymbol{I}_{\boldsymbol{D}}\boldsymbol{-}\boldsymbol{I}_{\boldsymbol{O}}} \right)$ | **Attributable Fraction (%)**  $\left[ \left( \boldsymbol{1-}\frac{\boldsymbol{I}_{\boldsymbol{o}}}{\boldsymbol{I}_{\boldsymbol{D}}} \right)\boldsymbol{\times100\%} \right]$ |
| --- | --- | --- | --- | --- | --- | --- |
| All groups | 254 (241, 268) | 164 (153, 175) | 1.55 (1.42, 1.69) | 90 (73, 108) | 1106 (892, 1320) | 36 (30, 41) |
| Birth registered in New South Wales | 266 (250, 281) | 177 (164, 191) | 1.50 (1.36, 1.64) | 88 (68, 109) | 1134 (872, 1396) | 33 (27, 40) |
| Birth registered in Western Australia | 217 (191, 242) | 115 (93, 137) | 1.88 (1.46, 2.30) | 102 (68, 135) | 983 (658, 1309) | 47 (35, 59) |
| Non-Aboriginal | 237 (223, 250) | 154 (144, 165) | 1.53 (1.40, 1.67) | 82 (65, 99) | 1215 (961, 1468) | 35 (29, 41) |
| Aboriginal | 493 (422, 563) | 288 (208, 368) | 1.71 (1.18, 2.25) | 205 (98, 311) | 488 (234, 742) | 42 (23, 60) |
| gestational age <=36 weeks | 248 (234, 261) | 157 (146, 168) | 1.58 (1.44, 1.72) | 91 (73, 109) | 1100 (887, 1313) | 37 (31, 42) |
| Parity >=3 | 517 (449, 585) | 316 (233, 399) | 1.64 (1.16, 2.12) | 201 (94, 308) | 497 (232, 762) | 39 (21, 57) |
| Mother born in Oceania excluding Australia | 288 (226, 349) | 171 (109, 232) | 1.69 (0.98, 2.39) | 117 (30, 204) | 854 (220, 1488) | 41 (16, 65) |
| Mother aged < 20 years | 352 (280, 425) | 249 (177, 320) | 1.42 (0.92, 1.92) | 104 (2, 206) | 963 (18, 1908) | 29 (04, 54) |
| Mother smoked during pregnancy | 329 (293, 365) | 195 (159, 232) | 1.69 (1.32, 2.05) | 134 (83, 185) | 746 (460, 1031) | 41 (28, 54) |
| At least 1 dose within the 1st year of life | 235 (222, 249) | 166 (154, 177) | 1.42 (1.29, 1.55) | 70 (52, 87) | 1437 (1070, 1804) | 30 (23, 36) |

^a^ Rate per 100,00 infants followed from 39 days to 1 year of age.

^b^ Created by applying stabilised weights based on the inverse probability of treatment (IPT) methodology^4,6^ to adjust the demographic and risk characteristics in the ‘on-time’ group to match the ‘delayed’ group.

References

1. Greifer N, Stuart EA. Choosing the Estimand When Matching or Weighting in Observational Studies. arXiv preprint 2021; arXiv:2106.10577.

2. Pink B. Information paper: an introduction to socio-economic indexes for areas (SEIFA), 2006. Canberra: Australian Bureau of Statistics; 2008.

3. Hugo Centre for Migration and Population Research. The Accessibility/Remoteness Index of Australia, 2011. <https://www.adelaide.edu.au/hugo-centre/services/aria> (accessed 10 July, 2021).

4 Hernán MA, Robins JM. Causal Inference: What If. Boco Raton: Chapman & Hall/CRC; 2020.

5. Schacht A, Bogaerts K, Bluhmki E, Lesaffre E. A new nonparametric approach for baseline covariate adjustment for two‐group comparative studies. Biometrics. 2008; 64: 1110-1116.

6. Austin PC. An introduction to propensity score methods for reducing the effects of confounding in observational studies. Multivariate Behavioral Research. 2011; 46: 399-424.
